# Distinct Effects of Contraction Agonists on the Phosphorylation State of Cofilin in Pulmonary Artery Smooth Muscle

**DOI:** 10.1155/2008/362741

**Published:** 2007-11-06

**Authors:** Yan-Ping Dai, Shaner Bongalon, Violeta N. Mutafova-Yambolieva, Ilia A. Yamboliev

**Affiliations:** ^1^Department of Pharmacology, Center of Biomedical Research Excellence, University of Nevada School of Medicine, Reno, NV 89557, USA; ^2^Department of Physiology and Cell Biology, University of Nevada School of Medicine, Reno, NV 89557, USA

## Abstract

We hypothesized that agonist-induced contraction correlates with the phospho-cofilin/cofilin (P-CF/CF) ratio in pulmonary artery (PA) rings and cultured smooth muscle cells (PASMCs). PA rings were used for isometric contractions and along with PASMCs for assay of P-CF/CF by isoelectric focusing and immunoblotting. The P-CF/CF measured 22.5% in PA and differentiated PASMCs, but only 14.8% in undifferentiated PASMCs. With comparable contraction responses in PA, endothelin-1 (100 nM) and norepinephrine (1 *μ*M) induced a 2-fold increase of P-CF/CF, while angiotensin II (1 *μ*M) induced none. All agonists activated Rho-kinase and LIMK2, and activation was eliminated by inhibition of Rho-kinase. Microcystin LF (20 nM) potentiated the angiotensin II, but not the 5-hydroxytryptamine (1 *μ*M)-mediated increase of P-CF/CF. In conclusion, all tested agonists activate the Rho-kinase-LIMK pathway and increase P-CF/CF. Angiotensin II activates PP2A and counteracts the LIMK-mediated CF phosphorylation. CF phosphorylation stabilizes peripheral actin structures and may contribute to the maximal contraction of PA.

## 1. INTRODUCTION

The pulmonary circulation facilitates gas exchange between inhaled air and blood, and serves as a blood reservoir and a filter. Smooth muscle cell layers of pulmonary arteries (PA) play a primary role in these functions by regulating vascular myogenic tone and pulmonary blood flow. The majority of smooth muscle cells (SMCs) in healthy pulmonary blood vessels is differentiated, and their primary functions are to contract and relax. These functions are carried out by actin and myosin, which provide the driving force of the contraction response [1, 2]. However, the actomyosin coupling and production of muscle force is modulated by other proteins associated with the contraction domain that include the actin binding proteins calponin, h-caldesmon, and tropomyosin [3, 4]. It is also becoming increasingly clear that SMC contraction depends on proteins that associate with actin structures proximal to the cell membrane such as cSrc, focal adhesion kinase (FAK), paxillin, integrins, HSP27, and cofilin. These proteins participate in the formation of focal adhesion complexes by mediating attachment of stress fibers to the cell membrane [5].

Cofilin (CF) is an actin binding protein which functions in the cell periphery as the principal depolymerizing protein of subcortical actin complexes [6, 7]. CF localizes at the pointed ends of actin filaments and decreases their length by dissociating ADP-coupled actin monomers [8]. The actin depolymerization activity of CF is regulated by pH, association with adaptor proteins, and interaction with phosphoinositides [9, 10]. However, reversible phosphorylation is the most important mechanism: only nonphosphorylated CF possesses actin filament-depolymerizing activity, while Ser3-phosphorylated CF lacks such activity [11, 12]. Therefore, the phosphorylation state of CF governs its capacity to maintain the integrity of the subcortical actin network.

Phosphorylation of CF is mediated by two protein kinase families: the testicular protein kinase (TESK) and LIM kinase (LIMK). However, TESK is activated upon cell attachment onto fibronectin and transduces signals from integrin receptors to peripheral actin structures [13]. LIMK is activated by soluble ligands and activation is mediated by monomeric GTPases of the Rho family and subsequent activation of the Rho-dependent protein kinases PAK and Rho-kinase [14–16]. Rho GTPases can be activated by diverse agents that include contraction neurotransmitters [17], hormones [18], mitogens and growth factors [19–22], angiogenic agents [23], or cell stress [24–26]. Therefore, the Rho GTPase-Rho-kinase-LIMK pathway may provide the key signal that controls the CF-mediated remodeling of the subcortical actin network in response to activation of diverse cell membrane receptors or stress factors [11, 14]. However, the activation pattern and the role of CF for actin filament remodeling in contracting vascular smooth muscle is undefined.

The goal of this study is to evaluate how contraction agonists alter the phosphorylation state of cofilin in freshly dissected canine PA rings and in cultured PASMCs, and to assess signal transduction events involved in the cofilin phosphorylation at rest and during contraction of pulmonary artery smooth muscle.

## 2. MATERIAL AND METHODS

### 2.1. Animal treatment and tissue dissection

The investigation conforms to the *Guide for the Care and Use of Laboratory Animal* published by the US National Institutes of Health (NIH Publication no. 85-23, revised 1996), and with specific protocols approved by the Animal Care and Use Committee at the University of Nevada Reno. Canine lungs were obtained from adult mongrel dogs of either sex euthanized by barbiturate overdose. Lungs were dissected and second-order pulmonary artery branches were isolated, which were then used to cut rings for isometric contraction experiments, or to disperse and culture vascular myocytes, as described in our previous publications [27].

### 2.2. Dispersion of PASMCs

Second-order branches of canine PA were dissected, cleaned from connective tissue, and placed in Ca^2+^-free Hank's solution, containing 125 mM NaCl, 5.36 mM KCl, 15.5 mM NaHCO_3_, 0.34 mM Na_2_HPO_4_, 0.44 mM KH_2_PO_4_, 10 mM glucose, 2.9 mM sucrose, 10 mM HEPES, pH 7.4, at 37°C. Blood vessels were opened by longitudinal dissections, endothelial cells were scraped with cotton swabs and smooth muscle layers were minced and digested, and smooth muscle cells were cultured as described previously [27].

### 2.3. Isometric contraction experiments

Freshly dissected canine PA rings (about 2 mm diameter) were mounted in 10 ml organ baths and force displacements were monitored with Fort 10 isometric force transducers in a Myobath 4 system (World Precision Instruments, Sarasota, Fla, USA). A resting force of 1 g was applied to each muscle segment. This was found to stretch tissue segments to near the optimum length for tension development. In all experiments, tissues were initially equilibrated for 1 hour followed with at least 3 alternating 3-minute exposures to KCl (30 mmol/L) every 15 minutes in order to establish viability and equilibrate the tissue. Contraction agonists were added to bath solutions to a final concentration of 1 *μ*M norepinephrine (NE), angiotensin II (AngII) or 5-hydroxytryptamine (5-HT), 100 nM endothelin-1 (ET-1), or 2 U/ml thrombin. Contraction responses were monitored for 5 minutes, followed by extensive wash. In some experiments PA rings were incubated with Y-27632 (1 *μ*M) or microcystin LF (MCLF, 20 nM) for 1 hour prior to and during the incubation with contraction agonists. At the plateau of contraction, tissues were snap-frozen and homogenized to extract soluble protein.

### 2.4. Treatment of cultured PASMCs

First passage PASMCs were grown to confluence in complete growth medium M199 supplemented with 10% newborn calf serum (NCS) and then differentiated for three days in serum-free medium. Cells were then incubated for different times with 1 *μ*M NE, AngII or 5-HT, 100 nM ET-1, 2 U/ml thrombin. In some experiments, cells were preincubated for 1 hour with Y-27632 (1 *μ*M) or microcystin LF (MCLF, 20 nM) prior to incubation with contraction agonists.

### 2.5. Total protein extraction and immunoblotting

Total protein was extracted from PA rings by glass-glass homogenization in 10 volumes of Ripa buffer (50 mM Tris, pH 7.4, 150 mM NaCl, 5 mM Na_2_EDTA, 0.5% (v/v) NP40, 0.5% (v/v) Triton X-100, 1 mM NaF, 1 *μ*M leupeptin, and 1 *μ*M AEBSF) and sonication. Treated PASMCs were lysed with Ripa buffer. Total protein concentrations were assayed by a Micro BCA Protein Assay kit (Pierce Biotechnology, Rockford, Ill, USA). Equal amounts of total protein were resolved by SDS-PAGE and transferred onto nitrocellulose membranes for 1 hour at 24 V (Genie blotter, Idea Scientific Company, Minneapolis, Minn, USA). The membranes were blocked for 30 minutes with LI-COR blocking buffer (LI-COR, Inc., Lincoln, Neb, USA) and probed simultaneously with a primary monoclonal antibody against CF (Upstate Biotechnology, Lake Placid, NY, USA) and a primary rabbit polyclonal antibody against P-CF (Cytoskeleton, Denver, Colo, USA) diluted in LI-COR buffer. After removal of primary antibodies, membranes were incubated with two secondary antibodies: anti-rabbit, coupled to an infrared fluorescence marker with emission wavelength of 800 nm (IR800, Rockland Immunochemicals, Pa, USA), and anti-mouse, coupled to an infrared fluorescence marker with emission wavelength of 680 nm (Alexa Fluor 680, Molecular Probes, Ore, USA), diluted in LI-COR buffer. Double-color fluorescent images were obtained with an Odyssey scanner (LI-COR, Inc., Lincoln, Neb, USA). Immunoreactive band densities of P-CF were normalized to immunoreactive bands of CF and treatment-dependent changes were expressed relative to untreated controls.

### 2.6. Isoelectric focusing (IEF) electrophoresis

IEF gel slabs were casted in plastic cassettes purchased from BioRad (Hercules, Calif, USA), with gel compositions recommended by the manufacturer. The predicted isoelectric point (pI) of canine CF is 8.07 (Acc. DR105214), therefore equal volumes of ampholytes with pH ranges 6/8 and 7/9 were added to the gel mixture to a final concentration of 1%. Total protein extracts from PA rings or cultured PASMCs were mixed with glycerol and ampholytes (same pH range as in the gel composition) to final concentrations of 40% glycerol and 1% ampholytes. Samples were then loaded on IEF gels and protein was resolved by a stepwise voltage program (100 V for 60 minutes, 250 V for 60 minutes, and 500 V for 30 minutes) in 20 mM NaOH as cathode buffer and 7 mM phosphoric acid as anode buffer. Vertical protein-free strips (about 5 mm wide) were excised from the edge of each gel and divided into 1 cm-long segments. Segments were then soaked in 100 *μ*l distilled water for assay of the pH gradient throughout the gel. Protein from the IEF gel was then transferred onto nitrocellulose in 7 mM acetic acid as the transfer buffer for 1 hour at 24 V and 4°C (Genie blotter, Idea Scientific Company, Minneapolis, Minn, USA). Membranes were probed with CF antibodies and immunoreactive images were developed and quantified as described in the previous section. To dephosphorylate CF, we incubated protein extracts with calf intestinal alkaline phosphatase as recommended by the manufacturer (Promega Corp., Madison Wis, USA) prior to the IEF gel electrophoresis and immunoblotting.

### 2.7. LIMK2 immunoprecipitation and activity assay

Total protein extracts from control and contraction agonist treated canine PASMCs were precleared with protein A/G agarose plus beads and then LIMK2 was immunoprecipitated with a goat polyclonal antibody (C-19, Santa Cruz Biotechnology, Inc. Santa Cruz, Calif, USA). Immunoprecipitates were washed first with Ripa buffer and then with phosphorylation buffer (50 mM Tris-HCl, pH 7.8, 2 mM MgCl_2_, 0.5 mM EDTA, and 50 mM NaCl). LIMK2 activity was assayed by the phosphorylation of recombinant CF (Cytoskeleton, Inc., Denver, Colo, USA) in vitro in the presence of [*γ*-^32^ *P*]ATP at 30°C for 15 minutes. Protein was resolved by SDS-PAGE (15% acrylamide), gels were stained with Coomassie brilliant blue (CBB) and after distaining were exposed onto phosphorimaging screens. CF radioactive spots were visualized with a Personal Molecular Imager FX (BioRad, Hercules, Calif, USA) and normalized to the CBB-stained bands. Stimulus-specific LIMK2 activation was expressed relative to the kinase activity of untreated controls.

### 2.8. Statistical methods

Results are presented as the mean ± SD. The *n* values refer to the number of parallel experiments. Student's t-test for paired and unpaired data, or one-way ANOVA was applied to test for differences between treatment means, as appropriate. Values of *P* < .05 were considered statistically significant.

## 3. RESULTS

### 3.1. Assessment of CF charge isoforms in canine pulmonary artery smooth muscle

**Figure 1:**
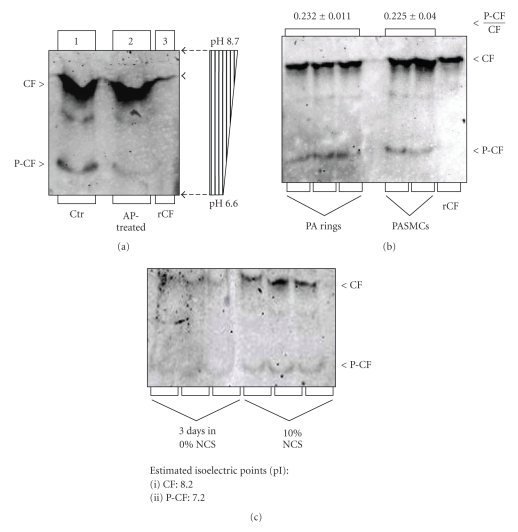
Assay of the phosphocofilin (P-CF) content in canine PA rings and cultured PASMCs by IEF. Total protein from freshly dissected PA rings (panel (a)) and undifferentiated and differentiated cells (panel (b)); recombinant CF control (rCF) were resolved by IEF electrophoresis on gels containing 1% of each ampholyte pH 6/8 and 7/9. For CF dephosphorylation, protein extracts were incubated with calf intestinal alkaline phosphatase (AP) prior to IEF electrophoresis. Protein was transferred onto nitrocellulose membranes and membranes were probed with a cofilin antibody that recognizes both unphosphorylated (CF) and phosphocofilin (P-CF). Immunoreactive bands were quantified by densitometry, and treatment-dependent changes of the P-CF/CF ratio were presented relative to untreated controls (Ctr). Vertical strips of gels (0.5 cm wide) were cut into 1 cm long segments and soaked in deionized water before assay of pH (pH bar in panel A). Panel (c): CF expression is upregulated in proliferating PASMCs (cultured in 10% NCS), compared to differentiated cells (cultured for 4 days in 0% NCS).

IEF electrophoresis and immunoblotting of total protein obtained from freshly dissected canine pulmonary arteries and cultured PASMCs visualized two strong and one fainter CF-like immunoreactive bands (Figure 1).One of the strong immunoreactive bands comingrated with bacterial recombinant CF (rCF, Figure 1(a), lane 3) and was thus identified as the unphosphorylated endogenous CF. To identify P-CF band we dephosphorylated endogenous P-CF by incubation with calf intestinal alkaline phosphatase (AP) and then immunoblotted AP-treated samples along with untreated control protein extracts. Incubation with AP significantly decreased the density of the second strongest immunoreactive band, which was identified as the endogenous P-CF (Figure 1(a), lane 2). pH measurements of extracts obtained by soaking IEF gel segments in distilled water, revealed a linear pH gradient (Figure 1(a), pH bar) and demonstrated that recombinant bacterial CF (unphosphorylated, used as control) and endogenous unphosphorylated CF had an apparent pI of 8.2. The estimated pI of the more acidic P-CF was 7.2 (Figure 1(a)). Our experimental CF pI (8.2) is consistent with the predicted pI of canine cardiovascular, mouse, and human CF (pI 8.07, Acc. DR105214, NM760088 and NM005507, resp.), and with estimated pI values of CF and P-CF of other laboratories [28].

To assay the fraction of the phosphorylated CF (P-CF) compared to the total CF (i.e., the P-CF/CF ratio), we used canine freshly dissected pulmonary artery rings and cultured PASMCs differentiated for three days in serum-free culture medium (Figure 1(b)). Average data from four parallel experiments demonstrated that P-CF accounts for 23.2 ± 1.1% of the total CF pool in pulmonary arteries and 22.5 ± 4.4% in differentiated PASMCs.

Cultured PASMCs have been used in previous studies of our and other laboratories. To test how cell culturing affects the fraction of P-CF, we compared the P-CF/CF ratio of differentiated (0% NCS for three days) and in proliferating PASMCs maintained in complete culture medium (10% NCS). Results from three parallel experiments demonstrated that while the P-CF content changed insignificantly, the total CF content increased in proliferating PASMCs (Figure 1(c)). As a result, the P-CF/CF ratio decreased from 22.5 ± 4.2% in differentiated PASMCs to14.8 ± 4.1% in proliferating PASMCs. These results indicate that while the total protein content of CF is higher, the fraction of the phosphorylated CF is relatively lower in proliferating than in differentiated PASMCs or freshly dissected pulmonary artery rings. These results are consistent with the notion that more dynamic actin filament remodeling in proliferating PASMCs is associated by higher CF activity.

### 3.2. Modulation of CF phosphorylation by contraction agonists in canine pulmonary artery strips and cultured PASMCs

**Figure 2:**
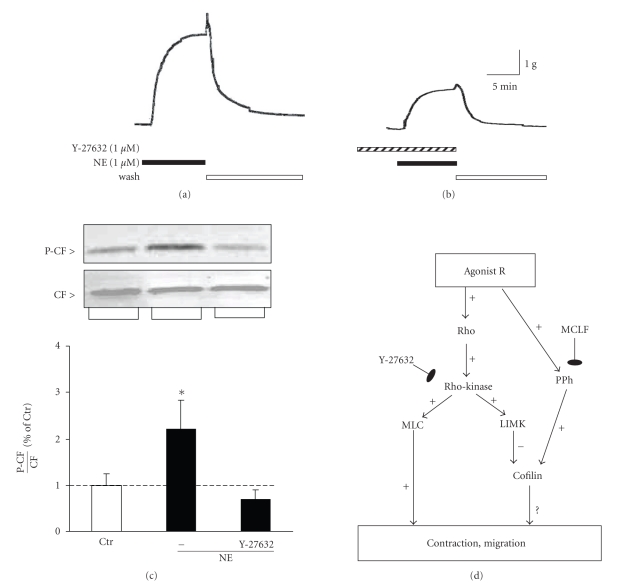
Norepinephrine-(NE) induced contraction and CF phosphorylation of PA rings were attenuated by the Rho-kinase inhibitor Y-27632. Freshly dissected canine PA rings were incubated with 1 *μ*M NE without (panel (a)) or after 1-hour preincubation with Y-27632 (1 *μ*M panel (b)). Strips were snap-frozen at the maximal contraction amplitude and homogenized. Total protein was resolved by SDS-PAGE and transferred onto nitrocellulose membranes. CF and P-CF were assayed by immunoblotting (panel (c)). Immunoreactive P-CF band densities (panel (c), top inset) were normalized to immunoreactive CF bands (panel (c), bottom inset), and treatment-induced changes in P-CF/CF ratio were expressed as percent of unstimulated controls (Ctr, bar graph). Panel (d): Schematic of signal transduction leading to phosphorylation of cofilin and documented CF-mediated cell functional responses. Abbreviations: Agonist R, agonist receptor; Rho, Rho GTPases; MLC, myosin light chain; LIMK, LIM kinase, PPh, protein phosphatases, MCLF, microcystin LF. Mean ± SD, (∗) *P* < .05 compared to untreated controls, *n* = 3.

To test whether smooth muscle contraction is associated with changes of the P-CF, we incubated freshly dissected pulmonary artery strips with NE and at the maximal amplitude we snap-froze tissues. Then we extracted and resolved total protein by SDS-PAGE, and assayed CF and P-CF by simultaneous immunoblotting using CF and P-CF antibodies, respectively. The NE-induced contraction was associated with increased phosphorylation of CF (Figures 2(a) and 2(c)). Incubation of PA rings with the Rho-kinase inhibitor Y-27632 prior to incubation with NE attenuated both the NE-induced contraction response (Figure 2(b)) and the NE-mediated increase of P-CF (Figure 2(c)). The reduction of the NE-stimulated mechanical response by Y-27632 is the result of inhibition of the Rho-kinase-dependent activation of myosin light chain (MLC), reported in previous studies [17, 29]. The reduction of the P-CF content, on the other hand, is the result of inhibition of the Rho-kinase-mediated activation of LIMK [30]. Thus, activation of adrenergic receptors is coupled to activation of Rho GTPases, Rho-kinase, and LIMK (Figure 2(d)).

**Figure 3:**
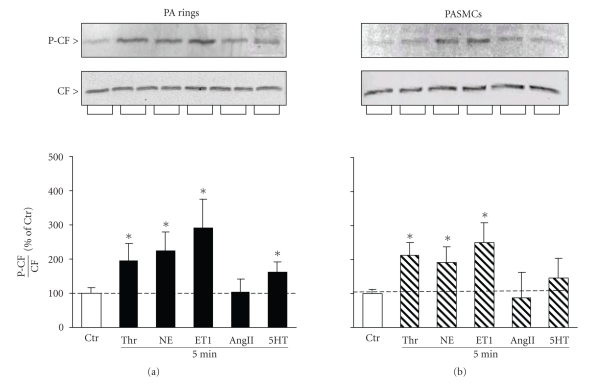
Contraction agonists stimulate cofilin phosphorylation in canine PA rings and cultured PASMCs. Freshly dissected PA rings and differentiated PASMCs were incubated with thrombin (Thr), norepinephrine (NE), endothelin-1 (ET1), angiotensin II (AngII), and 5-hydroxytryptamine (5-HT) for 5 minutes. Total protein was extracted and resolved by SDS-PAGE, and then transferred onto nitrocellulose. Membranes were probed simultaneously with CF and P-CF antibodies. Immunoreactive bands of P-CF were normalized to immunoreactive bands of CF (insets) and the agonist-induced changes in the P-CF/CF ratio were presented as percentage of untreated controls (Ctr, bar graphs). Mean ± SD, (∗) *P* < .05 compared to untreated controls, *n* = 4.

NE is the major contraction agonist in healthy blood vessels; however PASMCs are also exposed to other endogenous contraction agonists. To test how other contraction agonists affect P-CF, we incubated PA rings with ET-1, AngII, 5HT, and thrombin and then assayed the P-CF/CF ratio by immunoblotting. Similar to NE, these agonists induced contraction responses and the maximal contractions were partially inhibited by Y-27632 (not shown). However, these agonists had distinct effects on the P-CF/CF ratio: the ET-1 and thrombin-mediated contractions were associated with substantial about 2-fold increase of the P-CF/CF ratio, 5-HT stimulated an increase of 1.7-fold, while AngII caused no change of the CF phosphorylation (Figure 3(a)).

We also assayed the P-CF/CF ratio in PASMCs cultured in serum-free growth medium for 4 days prior to incubation with ET-1, AngII, 5-HT, and thrombin. Overall, the pattern of the P-CF/CF ratio in cultured PASMCs was reminiscent of the pattern in PA rings (Figure 3(b)). Together these results indicate that increase of the CF phosphorylation is not a common feature of all contraction agonists. The reduction of the mechanical responses by Y-27632 indicates that Rho-kinase-mediated signaling modulates the contraction to all contraction agents. Therefore, signal transduction mechanisms leading to changes of P-CF may dissociate downstream of Rho-kinase, or depend on activation of alternate pathways that counteract Rho-kinase-LIMK-mediated phosphorylation of CF.

### 3.3. Contraction agonists activate LIMK2

**Figure 4:**
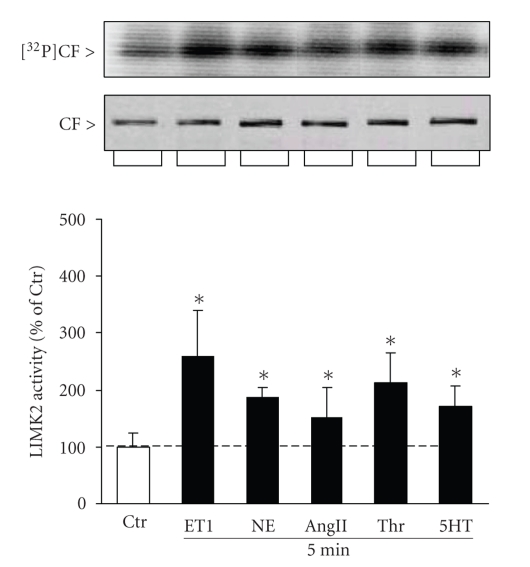
Contraction agonists activate LIMK2 in canine PASMCs. Canine PASMCs were incubated with thrombin (Thr), norepinephrine (NE), endothelin-1 (ET1), angiotensin II (AngII), and 5-hydroxytryptamine (5-HT) for 5 min. LIMK2 was immunoprecipitated from total protein homogenates and kinase activity was assayed by the phosphorylation of recombinant CF in the presence of [*γ*-^32^ *P*]ATP in vitro. Reaction protein was resolved by SDS-PAGE, gels were stained with Coomassie brilliant blue and then exposed on phosphorimaging screens. CF radioactive bands (top inset) were normalized to protein bands (lower inset) and the agonist-mediated activation of LIMK2 was expressed as percentage of the activity of untreated cell controls (Ctr, bar graph). Mean ± SD, (∗) *P* < .05 compared to untreated controls, *n* = 3.

LIMK2 is expressed in pulmonary artery smooth muscle cells [27] and is a direct target of Rho-kinases [31]. Because the tested contraction agonists activated Rho-kinase, it is likely that they activate also LIMK2. To test this possibility, we incubated canine PASMCs with NE, ET-1, AngII, thrombin, and 5-HT and then immunoprecipitated LIMK2 from cell total protein extracts. Kinase activity was assayed by the phosphorylation of recombinant CF in vitro in the presence of [*γ*-^32^ *P*]ATP. All contraction agonists activated LIMK2, with ET-1 being the strongest (261 ± 52%) and AngII being the weakest activator (125 ± 35%) at these concentrations (Figure 4). The activation of LIMK2 by ET-1, NE, 5-HT, and thrombin closely resembled the agonist-induced increase of the CF phosphorylation, indicating that activation of LIMK2 is directly coupled to CF phosphorylation (see Figure 3). However, the AngII-mediated activation of LIMK2 (Figure 4) caused insignificant change of the P-CF fraction (Figure 3), suggesting that in addition to the activation of LIMK2, the phosphorylation state of CF in AngII-treated cells may depend on other pathways as well.

### 3.4. PP2A is involved in the AngII-mediated phosphorylation of cofilin

**Figure 5:**
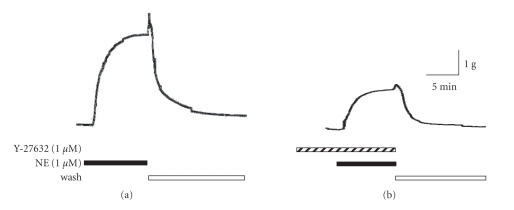
Inhibition of protein phosphatase PP2A potentiates AngII-induced phosphorylation of CF. Canine PASMCs were incubated with microcystin LF (MCLF, 20 nM) for 1 hour prior to 5 minutes incubations with angiotensin II (AngII, 1 mM) and 5-hydroxytryptamine (5-HT, 1 mM). Total protein was resolved by SDS-PAGE and then transferred onto nitrocellulose. Membranes were probed simultaneously with CF and P-CF antibodies. Immunoreactive bands of P-CF (a) were normalized to immunoreactive bands of CF (b) and the agonist-induced changes in the P-CF/CF ratio were presented as percentage of the ratio in untreated controls (Ctr, bar graphs). Mean ± SD, (∗) *P* < .05 compared to untreated controls, *n* = 3.

The AngII-mediated increase of P-CF was insignificant compared to NE, ET-1, 5-HT, and thrombin; therefore we tested the possibility that AngII activates CF phosphatases. Since PP2A dephosphorylates CF [12, 32], we incubated canine PASMCs with a PP2A inhibitor, microcystin LF (MCLF, 20 nM) for 1 hour prior to a 5-minute incubation with AngII and 5-HT (positive control). Incubation with MCLF alone caused a slight increase of the P-CF content to 132 ± 26% (*P* < .05) indicating that PP2A activity modulates the phosphorylation level of CF. Moreover, MCLF potentiated the AngII-stimulated CF phosphorylation from 104 ± 13% (AngII control, *P* > .05) to 198 ± 42% (AngII + MCLF, *P* < .05, *n* = 3) (Figure 5(a)). In the 5-HT-treated group, incubation with MCLF did not significantly potentiate the 5-HT-mediated increase of P-CF (Figure 5(b)). These results indicate that activation of AngII receptors is associated with activation of PP2A, which is likely to cause CF dephosphorylation in the AngII-treated group. However, activation of PP2A plays a minor role in the 5-HT-mediated phosphorylation of CF.

## 4. DISCUSSION

This study demonstrates that exposure of pulmonary artery smooth muscle to various contraction agonists is associated with stimulus-specific changes of the phosphorylation state of CF. CF functions primarily in cell subcortical areas and modulates the remodeling of peripheral actin structures and cellular responses to extracellular stimuli. During smooth muscle contraction, for example, phosphorylation and inactivation of CF could reduce dissociation of peripheral actin filaments and hence stabilize stress fiber attachment to focal adhesion complexes [30, 31]. Ultimately, this could facilitate a more efficient pull and potentiate cell shortening and development of maximal contraction. This scenario is consistent with the increased P-CF content in freshly dissected PA rings and in differentiated PASMCs exposed to NE, ET-1, 5-HT, and thrombin. It is also consistent with previous studies demonstrating that thrombin [33], 5-HT [34, 35], NE and ET-1 [34, 36] activate Rho GTPases and then Rho-kinase and LIMK. Surprisingly, however, the contraction response of PA to AngII developed without a concomitant increase of the P-CF content, although the AngII-induced contraction was associated with activation of Rho-kinase. We also confirmed that AngII, as well as ET-1, NE, thrombin, and 5-HT activate LIMK2 in our system, thus ruling out the possibility that the lack of change of the P-CF content is due to lack of activation of LIMK by AngII. Therefore, the phosphorylation of CF in AngII-treated pulmonary artery appeared to depend on other mechanisms as well.

The role of AngII in the activation of Rho GTPase has been controversial. Sakurada et al., for example, have reported lack of activation of Rho in rabbit aortic smooth muscle [34], however, AngII activates Rho in rat renal arterioles [37, 38]. These studies suggest that the AngII-mediated activation of the Rho GTPase- Rho-kinase-LIMK-CF pathway may vary among species and/or vascular beds. These differences may be underlined by differences in the relative expression of the two major AngII receptor types, that is, type I (AT1R) and type II (AT2R), or in their postreceptor mechanisms. Previous studies, for example, have demonstrated that activation of AT2R may negatively regulate RhoA and MLC phosphorylation [39]. Other studies have shown that AT2R is coupled to activation of the protein phosphatase PP2A [40, 41], which is also involved in the dephosphorylation of CF [12, 32]. Therefore, the effect of AngII on the phosphorylation of CF in PA smooth muscle may depend on the relative expression and activation of AT1R and AT2R and reflect the balance between the AT1R-mediated activation of the Rho-Rho-kinase-LIMK pathway and AT2R-mediated inhibition of Rho-Rho-kinase-LIMK signaling and/or activation of PP2A. Our results are consistent with the concept that AngII receptors mediate both activation of Rho-kinase and activation of a CF phosphatase such as PP2A. Recent studies point to slingshot as the primary CF phosphatase [42, 43], however, the link between AngII receptors and Slingshot remains undefined.

With respect to the functional role of cofilin in contracting vascular smooth muscle, our results are consistent with the following speculations. Firstly, CF phosphorylation and inactivation plays a minor, if any, role for the contraction response to NE, ET-1, thrombin, and 5-HT, and particularly to AngII. This conclusion does not exclude the possibility that inhibition of the CF phosphorylation may decrease the maximal contraction to NE, ET-1, thrombin, and 5-HT, or that inhibition of CF phosphatases may increase the maximal contraction to AngII. However, addressing these possibilities will require the development of specific pharmacological activators and inhibitors of CF kinases (i.e., LIMK and TESK) or CF phosphatases (i.e., Slingshot) for use in whole tissue preparations. Secondly, the P-CF content increases from a resting level of about 23% to 42% in thrombin-treated, and to about 50% in NE or ET-1-trated PA rings. Such accumulation of inactive P-CF has been shown to induce the formation of stress fibers and focal adhesions in HeLa cells [31]. Similarly, an increase of the P-CF/CF ratio in PA smooth muscle is likely to stabilize the peripheral actin network and focal adhesions, and be beneficial for the contraction response of smooth muscle cells.

## References

[1] Marston SB, Smith CWJ (1985). The thin filaments of smooth muscles. *Journal of Muscle Research and Cell Motility*.

[2] Marston SB, Burton D, Copeland O (1998). Structural interactions between actin, tropomyosin, caldesmon and calcium binding protein and the regulation
of smooth muscle thin filaments. *Acta Physiologica Scandinavica*.

[3] Durand-Arczynska W, Marmy N, Durand J (1993). Caldesmon, calponin and *α*-smooth muscle actin expression in subcultured smooth muscle cells from human airways. *Histochemistry and Cell Biology*.

[4] Halayko AJ, Salari H, Ma X, Stephens NL (1996). Markers of airway smooth muscle cell phenotype. *American Journal of Physiology*.

[5] Gerthoffer WT, Gunst SJ (2001). Invited review: focal adhesion and small heat shock proteins in the regulation of actin remodeling and
contractility in smooth muscle. *Journal of Applied Physiology*.

[6] Svitkina TM, Borisy GG (1999). Arp2/3 complex and actin depolymerizing factor/cofilin in dendritic organization and treadmilling of
actin filament array in lamellipodia. *Journal of Cell Biology*.

[7] DesMarais V, Ichetovkin I, Condeelis J, Hitchcock-DeGregori SE (2002). Spatial regulation of actin dynamics: a tropomyosin-free, actin-rich compartment of the leading edge. *Journal of Cell Science*.

[8] Palmgren S, Vartiainen M, Lappalainen P (2002). Twinfilin, a molecular mailman for actin monomers. *Journal of Cell Science*.

[9] Blondin L, Sapountzi V, Maciver SK, Lagarrigue E, Benyamin Y, Roustan C (2002). A structural basis for the pH-dependence of cofilin F-actin interactions. *European Journal of Biochemistry*.

[10] Ono S, Ono K (2002). Tropomyosin inhibits ADF/cofilin-dependent actin filament dynamics. *Journal of Cell Biology*.

[11] Edwards DC, Sanders LC, Bokoch GM, Gill GN (1999). Activation of LIM-kinase by Pak1 couples Rac/Cdc42 GTPase signalling to actin cytoskeletal dynamics. *Nature Cell Biology*.

[12] Ambach A, Saunus J, Konstandin M, Wesselborg S, Meuer SC, Samstag Y (2000). The serine phosphatases PP1 and PP2A associate with and activate the actin-binding protein cofilin
in human T lymphocytes. *European Journal of Immunology*.

[13] Toshima J, Toshima JY, Amano T, Yang N, Narumiya S, Mizuno K (2001). Cofilin phosphorylation by protein kinase testicular protein kinase 1 and its role in integrin-mediated actin
reorganization and focal adhesion formation. *Molecular Biology of the Cell*.

[14] Edwards DC, Gill GN (1999). Structural features of LIM kinase that control effects on the actin cytoskeleton. *Journal of Biological Chemistry*.

[15] Sumi T, Matsumoto K, Takai Y, Nakamura T (1999). Cofilin phosphorylation and actin cytoskeletal dynamics regulated by Rho- and Cdc42-activated LIM-kinase 2. *Journal of Cell Biology*.

[16] Sumi T, Matsumoto K, Shibuya A, Nakamura T (2001). Activation of LIM kinases by myotonic dystrophy kinase-related Cdc42-binding kinase *α*. *Journal of Biological Chemistry*.

[17] Pfitzer G, Arner A (1998). Involvement of small GTPases in the regulation of smooth muscle contraction. *Acta Physiologica Scandinavica*.

[18] Kuwahara K, Saito Y, Nakagawa O (1999). The effects of the selective ROCK inhibitor, Y27632, on ET-1-induced hypertrophic response in neonatal
rat cardiac myocytes—possible involvement of Rho/ROCK pathway in cardiac muscle cell hypertrophy. *FEBS Letters*.

[19] Nobes CD, Hawkins P, Stephens L, Hall A (1995). Activation of the small GTP-binding proteins rho and rac by growth factor receptors. *Journal of Cell Science*.

[20] Bornfeldt KE, Raines EW, Graves LM, Skinner MP, Krebs EG, Ross R (1995). Platelet-derived growth factor: distinct signal transduction pathways associated with migration versus proliferation. *Annals of the New York Academy of Sciences*.

[21] Dechert MA, Holder JM, Gerthoffer WT (2001). p21-activated kinase 1 participates in tracheal smooth muscle cell migration by signaling to p38 MAPK. *American Journal of Physiology*.

[22] Schmitz U, Thömmes K, Beier I, Vetter H (2002). Lysophosphatidic acid stimulates p21-activated kinase in vascular smooth muscle cells. *Biochemical and Biophysical Research Communications*.

[23] Keezer SM, Ivie SE, Krutzsch HC, Tandle A, Libutti SK, Roberts DD (2003). Angiogenesis inhibitors target the endothelial cell cytoskeleton through altered regulation of heat shock
protein 27 and cofilin. *Cancer Research*.

[24] Albinsson S, Nordström I, Hellstrand P (2004). Stretch of the vascular wall induces smooth muscle differentiation by promoting actin polymerization. *Journal of Biological Chemistry*.

[25] Yang X, Yu K, Hao Y (2004). LATS1 tumour suppressor affects cytokinesis by inhibiting LIMK1. *Nature Cell Biology*.

[26] Lee S, Helfman DM (2004). Cytoplasmic P21^Cip1^ is involved in Ras-induced inhibition of the ROCK/LIMK/Cofilin pathway. *Journal of Biological Chemistry*.

[27] Bongalon S, Dai Y-P, Singer CA, Yamboliev IA (2004). PDGF and IL-1*β* upregulate cofilin and LIMK2 in canine cultured pulmonary artery smooth muscle cells. *Journal of Vascular Research*.

[28] Verdijk P, van Veelen PA, de Ru AH (2004). Morphological changes during dendritic cell maturation correlate with cofilin activation and translocation to
the cell membrane. *European Journal of Immunology*.

[29] Kawano Y, Fukata Y, Oshiro N (1999). Phosphorylation of myosin-binding subunit (MBS) of myosin phosphatase by Rho-kinase in vivo. *Journal of Cell Biology*.

[30] Maekawa M, Ishizaki T, Boku S (1999). Signaling from Rho to the actin cytoskeleton through protein kinases ROCK and LIM-kinase. *Science*.

[31] Amano T, Tanabe K, Eto T, Narumiya S, Mizuno K (2001). LIM-kinase 2 induces formation of stress fibres, focal adhesions and membrane blebs, dependent on its activation
by Rho-associated kinase-catalysed phosphorylation at threonine-505. *Biochemical Journal*.

[32] Meberg PJ, Ono S, Minamide LS, Takahashi M, Bamburg JR (1998). Actin depolymerizing factor and cofilin phosphorylation dynamics: response to signals that regulate neurite extension. *Cell Motility and the Cytoskeleton*.

[33] Oriowo MA (2004). Chloride channels and *α*1-adrenoceptor-mediated pulmonary artery smooth muscle contraction:
effect of pulmonary hypertension. *European Journal of Pharmacology*.

[34] Sakurada S, Okamoto H, Takuwa N, Sugimoto N, Takuwa Y (2001). Rho activation in excitatory agonist-stimulated vascular smooth muscle. *American Journal of Physiology*.

[35] Kandabashi T, Shimokawa H, Mukai Y (2002). Involvement of Rho-kinase in agonists-induced contractions of arteriosclerotic human arteries. *Arteriosclerosis, Thrombosis, and Vascular Biology*.

[36] Channick RN, Sitbon O, Barst RJ, Manes A, Rubin LJ (2004). Endothelin receptor antagonists in pulmonary arterial hypertension. *Journal of the American College of Cardiology*.

[37] Nakamura A, Hayashi K, Ozawa Y (2003). Vessel- and vasoconstrictor-dependent role of Rho/Rho-kinase in renal microvascular tone. *Journal of Vascular Research*.

[38] Banes-Berceli AK, Shaw S, Ma G (2006). Effect of simvastatin on high glucose- and angiotensin II-induced activation of the JAK/STAT pathway in mesangial cells. *American Journal of Physiology*.

[39] Savoia C, Tabet F, Yao G, Schiffrin EL, Touyz RM (2005). Negative regulation of RhoA/Rho kinase by angiotensin II type 2 receptor in vascular smooth muscle cells:
role in angiotensin II-induced vasodilation in stroke-prone spontaneously hypertensive rats. *Journal of Hypertension*.

[40] Huang XC, Sumners C, Richards EM (1996). Angiotensin II stimulates protein phosphatase 2A activity in cultured neuronal cells via type 2 receptors in
a pertussis toxin sensitive fashion. *Advances in Experimental Medicine and Biology*.

[41] Moore SA, Huang N, Hinthong O, Andres RD, Grammatopoulos TN, Weyhenmeyer JA (2004). Human angiotensin II type-2 receptor inhibition of insulin-mediated ERK-2 activity via a G-protein
coupled signaling pathway. *Molecular Brain Research*.

[42] Niwa R, Nagata-Ohashi K, Takeichi M, Mizuno K, Uemura T (2002). Control of actin reorganization by Slingshot, a family of phosphatases that dephosphorylate ADF/cofilin. *Cell*.

[43] Ohta Y, Kousaka K, Nagata-Ohashi K (2003). Differential activities, subcellular distribution and tissue expression patterns of three members of Slingshot family
phosphatases that dephosphorylate cofilin. *Genes to Cells*.

